# The association between plasma proneurotensin and glucose regulation is modified by country of birth

**DOI:** 10.1038/s41598-019-50040-3

**Published:** 2019-09-20

**Authors:** A. Fawad, P. M. Nilsson, J. Struck, A. Bergmann, O. Melander, L. Bennet

**Affiliations:** 1 Department of Clinical Sciences, Lund University, Malmö, Sweden; 2 Sphingotec GmbH, Hennigsdorf, Germany; 3 Waltraut Bergmann Foundation, Hohen Neuendorf, Germany; 4 Department of Internal Medicine, Skåne University Hospital, Malmö, Sweden; 5 Metabolic Center, Region Skåne, Malmö, Sweden; 6 Center for Primary Health Care Research, Region Skåne, Malmö, Sweden

**Keywords:** Type 2 diabetes, Obesity

## Abstract

The prevalence of type 2 diabetes (T2D) has increased dramatically in Middle Eastern populations that represent the largest non-European immigrant group in Sweden today. As proneurotensin predicts T2D, the aim of this study was to investigate differences in proneurotensin levels across populations of Middle Eastern and Caucasian origin and to study its associations with indices of glucose regulation. Participants in the age 30 to 75 years, living in Malmö, Sweden, and born in Iraq or Sweden, were recruited from the census register. Anthropometrics and fasting samples were collected and oral glucose tolerance tests conducted assessing insulin secretion (DIo) as well as insulin sensitivity (ISI). A total of 2155 individuals participated in the study, 1398 were Iraqi-born and 757 were Swedish-born participants. Higher fasting proneurotensin levels were observed in Iraqi- compared to Swedish-born participants (137.5 vs. 119.8 pmol/L; p < 0.001) data adjusted for age, sex and body mass index. In Iraqi participants only, plasma proneurotensin was associated with impaired glucose regulation assessed as ISI, DIo and HbA_1c_, and significant interactions between country of birth and proneurotensin were observed (*P*_*interaction* ISI_ = 0.048; *P*_*interaction DIo*_ = 0.014; P_*interaction*HbA1c_ = 0.029). We report higher levels of proneurotensin in the general Middle Eastern population. The finding that Middle Eastern origin modifies the relationship of proneurotensin with indices of glucose regulation suggests that proneurotensin may be a stronger determinant of T2D in Middle Eastern as compared to Caucasian populations. These findings may explain part of the excess T2D risk in the Middle Eastern population but needs to be explored further.

## Introduction

Type 2 diabetes (T2D) is one of the most challenging health problem of the 21st century. It has affected 317 million people but its prevalence is still increasing rapidly. Urbanization and migration are known risk factors for T2D^1^. In the past few decades, millions of people have left their homelands and migrated to Europe due to political instability. Immigrants from Iraq represent the largest non-European immigrant group in Sweden today. The population-based MEDIM study has previously shown that a Middle Eastern immigrant population in southern Sweden has twice as high prevalence rate of T2D as compared to the native Swedish background population, and thus at high risk of developing T2D^2^. The excess risk can partly, however not fully, be explained by high prevalence of known cardiometabolic risk factors such as obesity, sedentary lifestyle and family history of diabetes that cluster in this population^2^. Further, as shown in the MEDIM study, insulin resistance is more pronounced and more strongly associated with T2D in the Iraqi immigrant population than in the Swedish born population. The comparatively high insulin resistance in Iraqi immigrant population may partly be explained by food culture. Over 70 percent of the Iraqi immigrant population regularly eat traditional dishes, despite living in Sweden for more than two decades. These traditional dishes contain high percentage of fat, that can contribute to insulin resistance and also influence cardiometabolic risk biomarkers^3^. It is still unclear whether diet composition affects insulin sensitivity and biomarkers of inflammation through its primary effect on body weight and adiposity, or whether it is the specific types and patterns of food consumed that directly affects these intermediate disease risk biomarkers.

Although T2D results from metabolic defects in insulin secretion and insulin sensitivity, most of the approximately 120 genetic T2D risk loci identified to date are related to beta cell dysfunction and impaired insulin secretion^4^. However irrespective of study design, participant’s race or ethnicity, the addition of genome-wide association markers into conventional risk models have produced little improvement in predicting DM2^5^. However, there are humoral predictors of T2D that may be associated with ethnic-dependent genetic risk. For instance in the Turkish population, studies have shown that single nucleotide polymorphisms (SNPs) in the adipose tissue specific protein encoded by the adiponectin gene, modulates insulin sensitivity and contributes to increased risk of DM2^6^.

Neurotensin is a 13 amino acid peptide, which is found in the gastrointestinal, central nervous and cardiovascular systems^7,8^. In the gut, neurotensin is released after fat intake and contributes to intestinal fat absorption and metabolism^9^. Neurotensin is unstable *in vivo* and *in vitro*, but by using an immunoassay, we can detect the stable precursor hormone proneurotensin, which in turn converts to mature neurotensin^9–11^. Recent animal studies have shown that a high-fat diet contributes to elevated neurotensin secretion^7^. Neurotensin knock-out mice (i.e. lacking neurotensin) displayed reduced intestinal fat absorption and abdominal fat accumulation and hence they were substantially protected against diet-induced obesity, insulin resistance and liver steatosis^9^. In dysmetabolic conditions in humans, a linear association is observed between plasma proneurotensin, and parameters related to glucose metabolism impairment such as hepatic damage in non-alcoholic fatty liver disease (NAFLD)^12^. In large, prospective population-based studies of Caucasians, elevated plasma proneurotensin levels are strong and independent predictor of incident obesity, T2D, cardiovascular disease and premature mortality^9–11,13^.

In this observational study, we hypothesized that proneurotensin may contribute to impaired glucose regulation and that the effect may differ across populations of Middle Eastern and Caucasian origins. Thus, our aim was to compare the plasma proneurotensin levels and further to investigate the associations between proneurotensin and glucose regulation (assessed as insulin secretion, insulin action and HbA_1c_) between populations of Middle Eastern (Iraqi) and Caucasian (Swedish) origin. To the best of our knowledge, studies investigating effect of plasma proneurotensin on glucose regulation comparing populations of different origin, in whom both diet and genetic backgrounds most likely differ, have not been previously explored.

## Methods

### Design, setting and subjects

As previously described, citizens of Malmö born in Iraq or Sweden, aged between 30 to 75 years of age, were randomly selected from the census register and invited by mail and phone to participate in this population-based survey^14^. We recruited participants living in the same geographical area, matched for sex and age-distributions. Study participants were grouped into those born in Iraq (first-generation Iraqi born immigrants) and those born in Sweden^14^. The median time for the Iraqi immigrant population living in Sweden was 17 years (interquartile range 12–23 years)^15^. No participants born in Sweden had parents born in Iraq. All citizens in Sweden have access to the same public financed system with minimum fees around 200 SEK/visit.

We excluded people with severe physical disabilities or mental illness. Examinations were conducted within a relatively short time-frame (February 1st, 2010, through December 31st, 2012) to minimize assessment biases and cohort effects. A flow chart of the recruitment has been previously published^16^.

#### Physical examination

Clinical variables such as blood pressure, height, weight, waist circumferences, and BMI were performed and assessed by trained Swedish- and Arabic-speaking research nurses as described previously^14^.

#### Questionnaires

Information of lifestyle, comorbidity, current medications, family history of diabetes (in biological parents and/or siblings), and socio-demography was collected through interviews by Arabic- and Swedish-speaking nurses using structured questionnaires in Swedish and Arabic^14^. The questionnaires were translated and back-translated by two independent professional translators with Arabic as their native language^14^.

#### Blood samples, oral glucose tolerance test and matsuda indices

Participants were instructed not to eat or drink anything except water after 10 p.m. in the evening before testing. Fasting blood samples were collected prior to a 75-g oral glucose tolerance test (OGTT).

During OGTT, blood samples were collected at 0, 30, 60, 90 and 120 min measuring plasma glucose and serum insulin levels. Immediately after sampling, blood glucose levels were measured in fresh plasma from venous blood, using a photometer (HemoCue AB, Ängelholm, Sweden) as described previously^17^. Serum insulin levels were assessed using a radioimmunoassay (Access^©^ Ultrasensitive Insulin, Beckman Coulter, USA)^18^. Plasma triglycerides (p-TG), high-density lipoprotein cholesterol (p-HDL) and low-density-lipoprotein cholesterol (p-LDL-C) levels were determined as previously described^17^.

Matsuda indices assessing insulin sensitivity index (ISI), corrected insulin response (CIR) and oral disposition index (DIo) were calculated from the OGTT results as previously described^14,19–22^.

Plasma proneurotensin was measured from stored fasting plasma, frozen at −80 °C and stored immediately after sampling. Assays were performed blinded to clinical data at an independent laboratory (ICI immunochemical Intelligence GmbH, Berlin, Germany) by using a one-step sandwich immunoassay based on a chemiluminescence label and coated-tube technique (SphingoTec^©^, Hennigsdorf, Germany). The assay had a functional sensitivity of 10 pmol/L determined as the lowest concentration measurable with an inter-assay precision of maximally% coefficient of variation. The limit of detection of proneurotensin precursor fragment was 1.9 pmol/L^10^. Plasma proneurotensin in the Swedish-born group was further categorised into tertiles, i.e. three equally large groups:

- First tertile proneurotensin <99.9 pmol/L.
- Second tertile proneurotensin ≥99.9 pmol/L to <145.3 pmol/L.
- Third tertile proneurotensin >145.3 pmol/L.

The Iraqi born participants were then categorised into the corresponding groups (i.e. with the same cut-off levels as the native Swedish population), Table 4.

Fatty liver index (FLI) is a proxy for Non-Alcohol Fatty Liver Disease (NAFLD), which is a strong risk factor for insulin resistance and T2D^23^, and was calculated as previously described by Balkau *et al*.:^24^ FLI = e^L^/(1 + e^L^) × 100, where L = 0.953 (log_e_ p-triglycerides) + 0.139 × BMI + 0.718 (log_e_ (GGT) + 0.053 × waist circumference −15.745.

#### T2D, family history of cardiometabolic disease

Participants with fasting plasma glucose levels of ≥7.0 mmol/L and/or by 2-h plasma glucose levels of ≥11.1 mmol/L were considered as new cases of T2D^25^. The OGTT was repeated on a separate day within two weeks, if only one glucose value was pathologic. For T2D diagnosis two plasma glucose values exceeding these thresholds were required^25^. Participants did not undergo an OGTT if they had known diabetes confirmed by medication with oral hypoglycaemic agents and/or insulin.

Family history of diabetes was considered as the presence of diabetes in first-degree relatives, i.e. biological parents, siblings and/or children: classified as no family history (FH−), or with diabetes in >1 first-degree relatives (FH+)^14^.

Cardiovascular disease was self-reported and included history of angina pectoris, myocardial infarction and/or stroke.

### Ethical approval and consent to participate

All participants have provided written informed consent to participate. The Ethics Committee at Lund University approved the study (application nos. 2009/36 and 2010/561). This investigation conforms to the principles outlined in the Declaration of Helsinki^26^.

### Consent for publication

The data does not include any individual personal data including individual details, images or videos and hence consent for publication is not applicable in this study.

## Statistics

### Statistical analysis

Analyses were performed using SPSS Statistics 23. After age- and sex-adjustments, least squares means were derived assessing linear regression. Differences in proportions were assessed by logistic regression adjusting for age and sex (Table 1). To approximate normal distributions, skewed variables (proneurotensin, insulin sensitivity index, and disposition index) were log_10_-transformed before analysis (Table 2). Associations between proneurotensin and insulin sensitivity index (ISI), proneurotensin and disposition index (DIo) and proneurotensin and HbA_1c_ respectively were estimated using multivariate linear regression analysis (Table 2). These data are expressed as β-coefficients with 95% confidence intervals, CI (Table 2). Associations with T2D were expressed as odds ratios (OR) with 95% confidence intervals (CI’s) (Table 3). In these models (Tables 2, 3), continuous independent variables were standardized to one unit variance (per 1 standard deviation, SD), in the strata of ethnicity and sex (z-scores). Correlation coefficients were assessed using Spearman’s test. We also studied associations between T2D and tertiles of proneurotensin, expressed as OR with 95% CI (Table 4). Interaction terms between proneurotensin and country of birth and between proneurotensin and gender were assessed for Tables 2–4.

**Table 1:** Characteristics of participants in the MEDIM study, born in Iraq or in Sweden.

	Born in Iraq *N* = 1398	Born in Sweden *N* = 757	*p*
Age, years	46.2 (9.6)	49.5 (11.2)	<0.001
Male sex, *n* (%)	819 (58.6)	378 (49.9)	0.010
Proneurotensin, pmol/L^a^	137.5 (111.1–164.7)	119.8 (99.9–145.3)	<0.001
Body mass index, kg/m^2^	29.3 (4.5)	27.3 (4.7)	<0.001
Waist circumference in men, cm	99.3 (10.6)	93.1 (10.9)	0.002
Waist circumference in women, cm	97.8 (11.7)	89.2 (14.1)	<0.001
Systolic Blood Pressure, mmHg^b^	127 (15.6)	134 (19.1)	<0.001
Diastolic Blood Pressure, mmHg^b^	77 (9.8)	80 (11.4)	<0.001
HbA_1c_ mmol/mol	37.9 (9.9)	36.3 (8.1)	<0.001
HbA_1c_%	4.6 (0.9)	4.5 (0.8)	<0.001
Plasma Low Density Lipoprotein (LDL) Cholesterol, mmol/L^c^	3.2 (0.8)	3.4 (0.9)	<0.001
Plasma Triglycerides, mmol/L^c^	1.6 (1.0)	1.2 (0.8)	<0.001
Insulin Sensitivity Index^a^	76.9 (58.4–101.1)	102.3 (77.7–133.0)	<0.001
Corrected Insulin Response^a,d^	167.8 (116.0–237.4)	140.1 (100.1–196.7)	<0.001
Disposition Index^a,d^	12413.3 (8316.3–18692.7)	13805.9 (9560.7–20150.4)	0.028
Type 2 diabetes, %	162 (11.6)	44 (5.8)	<0.001
Cardiovascular Disease, %	54 (3.9)	41 (5.4)	0.058
Family History of type 2 diabetes, %	716 (51.2)	200 (26.4)	<0.001

Data are presented as mean (standard deviations, SD), numbers (%) or medians^a^ (inter-quartile range, IQR).

^b^Including participants without blood pressure lowering medication

^c^Including participants without lipid lowering medication

^d^Cases where the glucose level at 30 min was > 4.44 mmol/l, and greater than the fasting glucose level, were included in the analysis^21^.

Differences in means between groups were studied using linear regression models adjusted for age and sex while differences in proportion of males between groups were studied using the chi-square test. Differences in proportions between groups were studied using logistic regression adjusted for age and sex. All tests were two-sided and a *p*-value of < 0.05 was considered statistically significant.

**Table 2:** Associations between insulin action (insulin sensitivity index), insulin secretion (disposition index) and HbA_1c_ as dependent variables and proneurotensin as the independent variable.

Proneurotensin^a^								*P*_Interaction_ ProNT^a^ *Country of Birth
Dependent variable	Model	Total study population		Born in Iraq		Born in Sweden		
		β	95% CI	β	95% CI	β	95% CI	
Insulin Sensitivity Index^a^								
	Model I	**−0.019**	**−0.032 to −0.007**	**−0.029**	**−0.044 to −0.014**	**−0.005**	**−0.025 to 0.015**	
	Model II	**−0.020**	**−0.032 to −0.008**					0.057
	Model III	**−0.017**	**−0.028 to −0.007**	**−0.026**	**−0.040 to −0.013**	−0.004	−0.021 to 0.012	
	Model IV	**−0.018**	**−0.029 to −0.007**					**0.050**
	Model V	**−0.023**	**−0.033 to −0.014**	**−0.031**	**−0.043 to −0.018**	−0.010	−0.026 to 0.006	
	Model VI	**−0.023**	**−0.033 to −0.013**					**0.048**
Disposition Index^a,b^								
	Model I	−0.014	−0.033 to 0.005	**−0.033**	**−0.057 to −0.010**	0.019	−0.011 to 0.049	
	Model II	−0.014	−0.033 to 0.004					**0.008**
	Model III	−0.013	−0.031 to 0.005	**−0.032**	**−0.056 to −0.009**	0.020	−0.009 to 0.050	
	Model IV	−0.013	−0.032 to 0.005					**0.007**
	Model V	−0.015	−0.033 to 0.004	**−0.033**	**−0.057 to −0.009**	0.018	−0.013 to 0.048	
	Model VI	−0.015	−0.033 to 0.004					**0.014**
HbA_1c_								
	Model I	**0.749**	**0.038 to 1.120**	**1.08**	**0.06 to 1.59**	0.112	−0.366 to 0.589	
	Model II	**0.746**	**0.377 to 1.114**					**0.014**
	Model III	**0.729**	**0.362 to 1.096**	**1.07**	**0.572 to 1.574**	0.071	−0.397 to 0.539	
	Model IV	**0.723**	**0.359 to 1.088**					**0.010**
	Model V	**0.779**	**0.411 to 1.146**	**1.07**	**0.569 to 1.580**	0.194	−0.274 to 0.662	
	Model VI	**0.772**	**0.405 to 1.139**					**0.029**

Data was adjusted for anthropometrics and country of birth, Model I to Model VI. Associations are presented as beta-coefficients (β) and 95% confidence interval (CI). Significant associations are bolded. There were no significant interactions between proneurotensin and gender in the model (data not shown).

Model I: Proneurotensin^a,c^, age^c^ and sex.

Model II: Proneurotensin^a,c^, age^c^, sex and country of birth.

Model III: Proneurotensin^a,c^, age^c^, sex and BMI^c.^

Model IV: Proneurotensin^a,c^, age^c^, sex, BMI^c^ and country of birth.

Model V: Proneurotensin^a,c^, age^c^, sex, BMI^c^ and Fatty Liver Index (FLI).

Model VI: Proneurotensin^a,c^, age^c^, sex, BMI^c^, FLI and country of birth.

Continuous independent variables were standardized in the strata of country of birth and sex (z-scores).

^a^Base 10 logarithm; ^b^CIR and DIo only included cases where the glucose level at 30 min was >4.44 mmol/l and was greater than the fasting glucose level^21^. ^c^Regression coefficients (β and odds ratios respectively) for continuous independent variables in Model I to IV were standardized to a unit variance (per 1 standard deviation) in the strata of ethnicity and sex (z-scores).

**Table 3:** Associations between T2D and proneurotensin, with data adjusted for anthropometrics and lifestyle-related risk factors, Model I to Model IV.

Proneurotensin^a^								*P*_Interaction_ ProNT^a^ *Country of Birth
Dependent variable	Model	Total study population		Born in Iraq		Born in Sweden		
		OR	95% CI	OR	95% CI	OR	95% CI	
T2D								
	Model I	**1.481**	1.253 to 1.750	**1.470**	**1.218 to 1.773**	**1.541**	**1.054 to 2.252**	
	Model II	**1.483**	1.254 to 1.755					NS
	Model III	**1.523**	1.298 to 1.788	**1.453**	**1.204 to 1.755**	**1.535**	**1.047 to 2.251**	
	Model IV	**1.470**	1.241 to 1.741					NS
	Model V	**1.534**	1.282 to 1.835	**1.484**	**1.213 to 1.817**	**1.713**	**1.141 to 2.572**	
	Model VI	**1.531**	1.278 to 1.835					NS

Associations presented as odds ratios (OR) and 95% confidence interval (CI). Significant associations are bolded.

**Table 4:** Risk of T2D in relation to country of birth and tertiles of proneurotensin (tertiles) assessed by logistic regression presenting odds ratios (OR) with 95% confidence intervals (CI’s).

	Total study population			
		OR	95% CI	
Age, years per 1 SD		**2.44**	**2.05**	**2.90**
Male sex		**1.51**	**1.06**	**2.16**
BMI kg/m^2^, per 1 SD		**1.52**	**1.29**	**1.80**
Family History of diabetes		**1.90**	**1.33**	**2.71**
Proneurotensin				
- First tertile	Born in Sweden	Reference		
	Born in Iraq	**2.02**	**0.82**	**4.97**
- Second tertile	Born in Sweden	0.61	0.17	2.15
	Born in Iraq	**3.57**	**1.53**	**8.37**
- Third tertile	Born in Sweden	**3.49**	**1.41**	**8.64**
	Born in Iraq	**4.82**	**2.13**	**10.89**

No significant interactions were observed between proneurotensin and country of birth or between proneurotensin and gender in the model.

All tests were two-sided and a *p*-value of <0.05 was considered statistically significant.

## Results

### Characteristics of participants

Our final study population included 1398 subjects born in Iraq (819 men, participation rate (PR) 45.9% and 579 women, PR 52.1%) and 757 subjects born in Sweden (400 men, PR 32.2%, and 357 women, PR 31.8%). The clinical and biomedical characteristics of study subjects are presented in Table 1.

The level of proneurotensin as well as BMI were significantly higher amongst Iraqi-born as compared to Swedish-born participants, data adjusted for age and sex (Table 1). Higher BMI was positively associated with higher proneurotensin levels (β 0.004; 95%CI: 0.001–0.006; *P* = 0.016). However, Iraqi origin remained positively associated with proneurotensin in spite of adjustment for age, sex and BMI (β 0.123; 95%CI: 0.082 to 0.163).

As previously reported, ISI was lower and the insulin secretion was not sufficient to compensate for the impaired level of insulin sensitivity, as reflected by the lower disposition index (Dio) in the Iraqi-born as compared to Swedish-born participants^14^. As previously reported, studies have shown that the prevalence of T2D was higher in Iraqi-born as compared to Swedish-born participants^2^. However, we did not observe any significant difference in the prevalence of cardiovascular disease.

### Proneurotensin and glucose regulation

**Figure 1:**
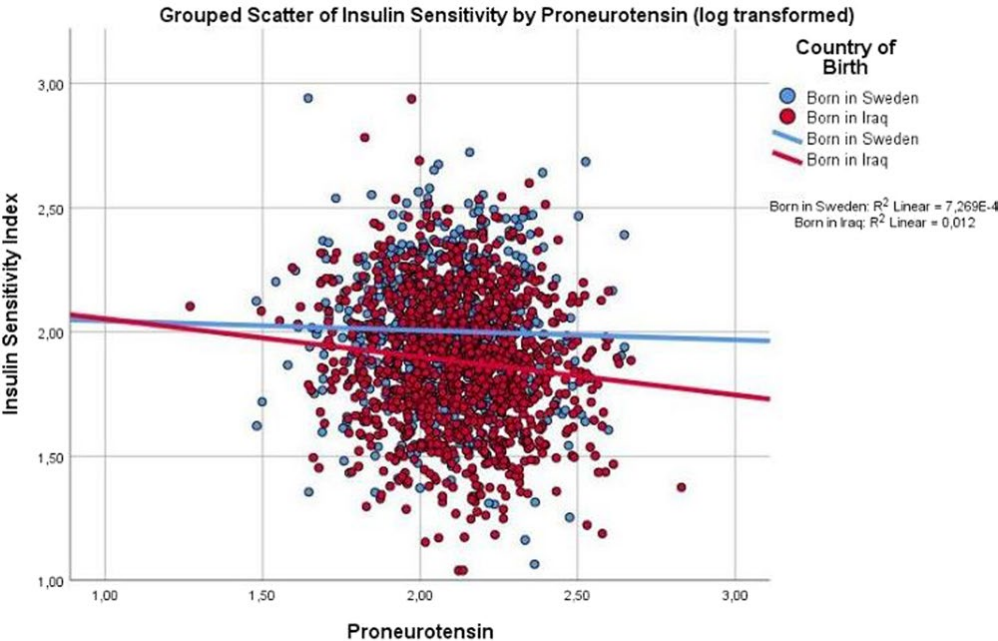
Scatter dot diagram with regression lines showing level of insulin action (insulin sensitivity index ISI) in relation to proneurotensin and country of birth. P < 0.001.

**Figure 2:**
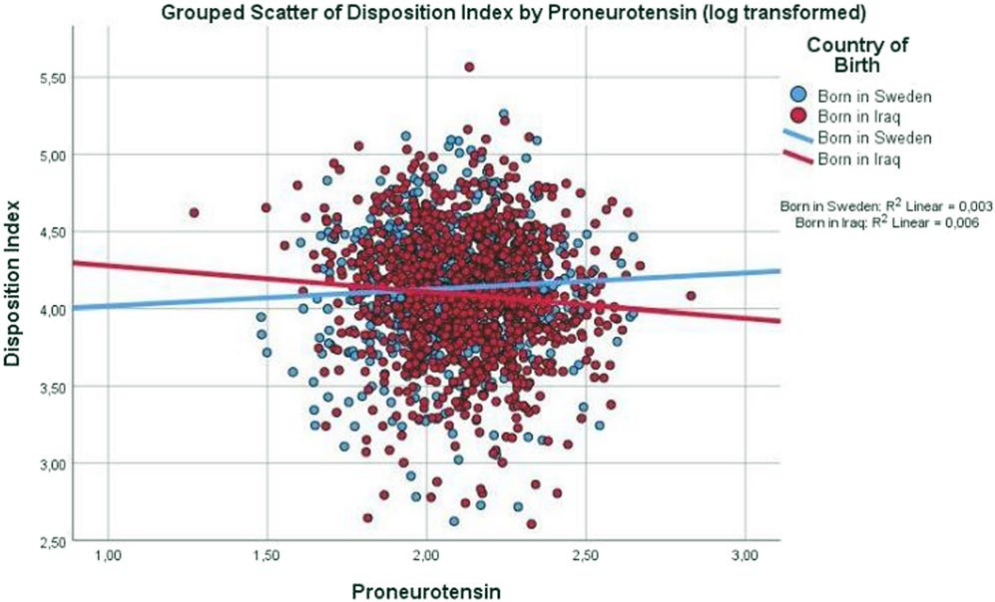
Scatter dot diagram with regression lines showing association between Disposition Index (DIo) in relation to proneurotensin and country of birth. P = 0.015.

**Figure 3:**
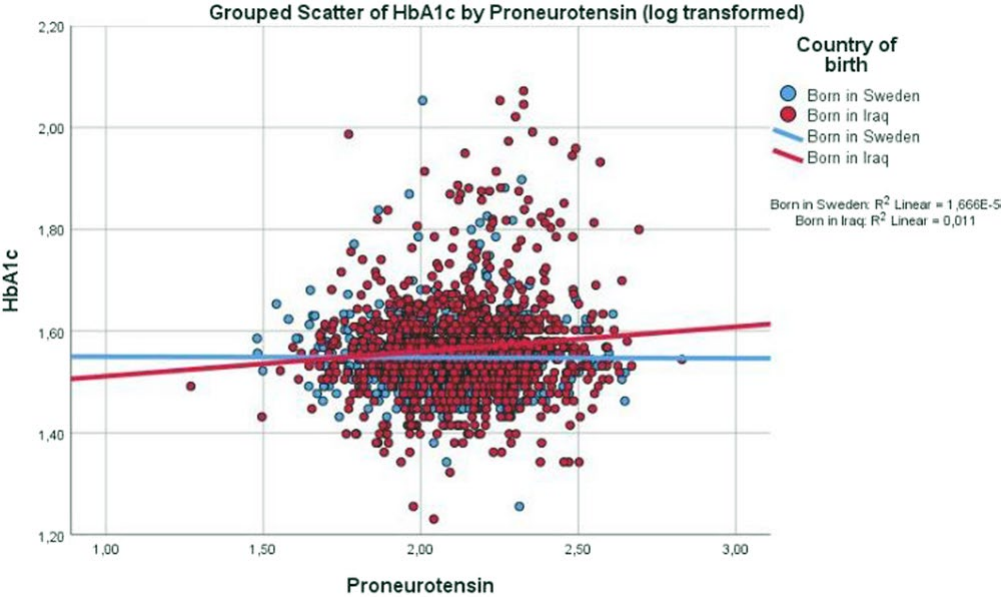
Scatter dot diagram with regression lines showing level of HbA1C in relation to proneurotensin and country of birth. P = 0.036.

Scatter dot diagrams in Figs 1–3 with regression lines present relationship between glucose regulation (assessed as insulin action (ISI), insulin secretion (DIo) and HbA_1c_, respectively), with proneurotensin (log transformed) in the Iraqi-born as compared to Swedish-born population. In the Iraqi-born population only, insulin action (ISI) and secretion showed significant negative correlations with proneurotensin (P_*ISI*_ < 0.001; P_*DIo*_ = 0.015), in Figs 1, 2, whereas HbA_1c_ showed a positive correlation with proneurotensin (P_HbA1c_ = 0.036), Fig. 3.

In a stepwise, multivariable linear regression analysis, Iraqi origin modified the relationship of proneurotensin with indices of glucose regulation as confirmed by interactions between country of birth and proneurotensin in all three models (P_*interaction ISI*_ = 0.048; P_*interaction DIo*_ = 0.014 and P_interaction HbA1c_ = 0.029), Table 2. In these models, ISI), DIo and HbA_1c_ respectively, were all associated with proneurotensin in the Iraqi-born population only. The models were adjusted for age, sex, anthropometrics and the fatty liver index. There were no significant interactions observed between gender and proneurotensin.

### Proneurotensin and diabetes risk

In a binary logistic regression analysis, the odds of T2D increased approximately by 1.5 fold per 1 unit increase (1 SD) of proneurotensin in both Iraqi- and Swedish-born participants (Table 3). The associations remained in the fully adjusted model. Further, Iraqi-born individuals within the third tertile displayed almost five times higher odds of T2D as compared to the reference of native Swedes within the first tertile of proneurotensin (Table 4). For each tertile, higher odds of T2D in Iraqis as compared to native Swedes were observed. This model did not, however have the power to detect any significant interactions between country of birth and proneurotensin, or between gender and proneurotensin.

## Discussion

Immigrants from the Middle East to Sweden are at high risk of obesity and T2D, but the exact factors contributing to this excess diabetes risk remain to be identified. In this population-based study addressing first-generation immigrants born in Iraq and living in Sweden, we hypothesized that elevated levels of the proneurotensin hormone associated with T2D risk^10,11^, may partly explain the increased T2D risk in the Iraqi born immigrant population. We here report significantly higher levels of proneurotensin in the Middle Eastern as compared to Swedish-born population. Further, we report a modifying effect of Middle Eastern origin on the association between proneurotensin and glucose regulation (assessed as insulin action, insulin secretion, or HbA_1c_). This implies that the effect of proneurotensin on glucose regulation is considerably stronger in populations of Middle Eastern as compared to Caucasian origin. This finding may contribute to the higher T2D risk in immigrants of Middle Eastern ancestry, but remains to be further studied.

Animal studies have shown that genetic knockout of neurotensin reduces diet-induced obesity, hepatic steatosis and insulin resistance in rodents^9^ which implies that high neurotensin may be causally related to cardiometabolic disease^9^. Genetic variations of the neurotensin receptor 3 (*NTSR3*) are linked to coronary artery disease^27,28^ and even *NTSR3* is suggested to regulate glucose transporter 4 (*GLUT4*)^29^ which is a key glucose transporter in peripheral muscle and adipose tissue regulating insulin sensitivity. Recent study results have shown that increased plasma neurotensin levels identifies the presence and severity of NAFLD in dysmetabolic individuals through insulin resistance related mechanisms^30^. These findings suggest a significant role of the proneurotensin system (neurotensin, *NTSR1* and *NTSR3)* on insulin resistance and glucose regulation contributing to the development of T2D^10^.

Previous data from Sweden and the US suggest that elevated plasma proneurotensin is a predictor of cardiovascular disease and T2D^10,11,13^ An intriguing interaction between proneurotensin-based prognostication and sex was observed where proneurotensin provided unique prognostic information for T2D, but only in women^13^. Our results in this study indicate comparably higher levels of plasma proneurotensin in Middle Eastern compared to Caucasian populations and that the effect of proneurotensin on glycaemic control differs across ethnicities. However, we could not confirm a sex-specific association of proneurotensin to diabetes as reported in the Malmö Preventive Project. A possible explanation could be that this is an observational study reporting an increased diabetes risk across populations of different origin in relation to proneurotensin. Another possible explanation could be that the statistical power of this study is not sufficient enough to illustrate the sex-specific associations as previously reported in Malmö Preventive Project, but only country of birth specific associations. Thus, we can conclude that neurotensin in groups of different origin has a higher impact on T2D risk than the differences in neurotensin between gender.

Middle Eastern immigrants to Sweden have previously been identified at high risk for obesity and T2D^2^. In the non-diabetic stage, this population has shown to be more insulin resistant and have higher mean HbA_1c_ levels as compared to the native Swedish population^14,31^. The poor glycaemic control is not fully explained by well-known risk factors for T2D such as differences in BMI, physical activity or other diabetes-related risk factors, but must be sought elsewhere^14^.

As high fat intake increases neurotensin secretion, a possible explanation to the higher levels of proneurotensin observed in the Iraqi as compared to the Swedish-born population, could be due to the high dietary intake of fat in the Iraqi-born population^3^. The diet alone, is not likely to explain the modifying effect of country of birth of proneurotensin on glucose regulation, as illustrated in Figs 1–3 showing that proneurotensin has a stronger association with insulin sensitivity, insulin action and HbA_1c_ in Iraqi subjects as compared to Swedes. One may hypothesize that there are country of birth associated differences in the efficacy of the processing from the non-cleaved proneurotensin (which is measured) to the mature and biologically hormone neurotensin, which in turn could explain the differences in these correlations, however, this remains speculative. It could also be speculated that altered sensitivity to the effects of neurotensin lies at the level of *NTSR1* and *NTSR3*, possibly due to genetic differences in Iraqi-born immigrants as compared to native Swedes, but this needs to be studied further.

### Strengths and limitations

The thorough sampling of cardiometabolic data including OGTT, performed in all subjects except in those with previously diagnosed T2D, is a strength of this study^14^. OGTT is a highly validated method, assessing insulin secretion and action by using Matsuda indices during a 2-h glucose load^19^. This method is correlated with hyperinsulinemic, euglycaemic clamp methodology which represents the gold standard technique for assessing insulin resistance^32^. Further, the influence of a potentially socioeconomic bias was reduced by recruiting participants from the same neighbourhood.

There are other possible hormones influencing diabetes risk in populations of different origin. However since previous population-based studies have shown that neurotensin has a strong impact on cardiometabolic disease this study focused on studying the role of neurotensin in a high-risk population for T2D. To the best of our knowledge the association of neurotensin with glucose regulation has not been previously studied in a Middle Eastern population.

A limitation is that the participation rate differed and was slightly higher in the Iraqis as compared to the Swedish-born population (approximately 40% vs. 30%). We have previously reported that there were no differences between participants and non-participants in prevalence of T2D, reflecting a representative study sample^2^. Our data showed corresponding levels of T2D in the Iraqi immigrant population with an Iraqi population still residing in Iraq^33^, further indicating the sample is representative for the Iraqi population in general. Although we could not fully account for residual confounding, the data was adjusted for the influence of several confounding factors.

Although causality between proneurotensin and T2D risk was previously shown in population-based, follow-up studies^10,11,13^, a possible limitation of this study is the cross-sectional study design, why we cannot draw any conclusions regarding causality.

## Conclusions

This observational study report higher levels of proneurotensin with a modifying effect of Middle Eastern ethnicity on glycaemic control. We conclude that this finding is likely to explain part of the excess T2D risk in this Middle Eastern compared to a native North-European population. Future studies focusing on causality and genetic variations within the neurotensin system across ethnicities could provide increased understanding of mechanisms involved contributing to the excess diabetes risk that the Middle Eastern population is exposed to.

## Data Availability

Data analysis are included in the published article and is available on request.
